# Effect of ambroxol on the concentration of cefotaxime in the bronchoalveolar lavage fluid of rats with pulmonary fibrosis

**DOI:** 10.3892/etm.2014.2112

**Published:** 2014-12-08

**Authors:** FENG CHEN, YUAN-XIA ZHANG, CAI-QING ZHANG

**Affiliations:** Department of Respiratory Medicine, Shandong Provincial Qianfoshan Hospital, Shandong University, Jinan, Shandong 250014, P.R. China

**Keywords:** pulmonary fibrosis, ambroxol, cefotaxime

## Abstract

This study aimed to investigate the effect of ambroxol on the concentration of cefotaxime in the bronchioalveolar lavage fluid of rats with bleomycin-induced pulmonary fibrosis. A total of 54 Wistar male rats were randomly divided into three groups, namely the normal control group, model group and ambroxol group. On experimental day 0, the rats were intratracheally instilled with bleomycin (5 mg/kg body weight) or sterile saline. The rats in the ambroxol group were then treated with ambroxol (35 mg/kg/day) intraperitoneally. On days 7, 14, 28 after instillation, six rats from each group were sacrificed, bronchial alveolar fluids were recovered and the lungs were collected for histopathological examination following the injection of cefotaxime (600 mg/kg) intravenously. The concentration of cefotaxime in the bronchial alveolar fluids was assayed by a liquid chromatography-mass spectrometry method. On day 7, the concentration of cefotaxime in the bronchial alveolar fluid of the ambroxol group was lower than that of the model group. On day 14, the concentration of cefotaxime in the bronchial alveolar fluids of the ambroxol group was higher than that of the model group, and the difference between these groups was significant statistically (P<0.001). On day 28, the concentration of cefotaxime in the bronchial alveolar fluids of the ambroxol group decreased sharply, and was lower than that of the model group (P=0.126). These results indicate that ambroxol increased the concentration of cefotaxime in the bronchial alveolar fluids at the primary fibrosis stage.

## Introduction

Idiopathic pulmonary fibrosis (IPF) is the most common specific form of idiopathic interstitial pneumonia. It is a chronic, progressive and irreversible lung disease with unknown cause and is usually fatal. Its histopathological or radiological appearance is similar to that typically observed for usual interstitial pneumonia (1–3). The mean survival time for diagnosed patients is 2.5 to 3.5 years (4); the survival period is short and the mortality rate is high. Ischemic heart disease, heart failure, bronchial cancer, infection and pulmonary embolism are significant causes of mortality in patients with IPF (5–8).

IPF complicated by infection can cause respiratory function to decline (4), which may lead to exacerbations and further reduce the arterial oxygen pressure. Active control of infection should help to improve lung function and reduce mortality.

The success of antibiotic therapy not only depends on the sensitivity of pathogenic microorganisms, but also the drug concentration in infected tissues, which is particularly important in the treatment of respiratory tract infections (9). Therefore, improving the concentration of antimicrobial drugs in the bronchial-pulmonary tissues is likely to help in controlling infection. According to previous studies, ambroxol increases the concentration of amoxicillin, erythromycin, ampicillin and other antibacterial drugs in the normal lung tissues of animals and humans (9–11). If ambroxol is able to improve antimicrobial concentrations in the lung tissue of patients with pulmonary fibrosis, treatment efficacy may be improved. However, to the best of our knowledge, whether ambroxol has an impact on the antimicrobial drug concentration in pulmonary interstitial fibrosis has not previously been reported. Bronchoalveolar lavage techniques are suitable for detecting the concentrations of antimicrobial agents in lung tissues (10). In the present study, the impact of ambroxol on the concentration of cefotaxime in the lung tissue of rats with pulmonary fibrosis was tested by a bronchoalveolar lavage technique to evaluate the role of ambroxol.

## Materials and methods

### Animal grouping and model preparation

A total of 54 male Wistar rats (clean grade) with weights of 180–220 g were purchased from the Animal Center of Shandong University (Jinan, China). The animals were randomly divided into three groups. These were a normal control group (group A), model group (group B) and ambroxol hydrochloride group (group C). The rats of the B and C groups were administered an intratracheal injection of bleomycin (5 mg/kg), and those of group A were administered an infusion of saline via the trachea. In addition, a daily intraperitoneal injection of ambroxol hydrochloride 35 mg/kg (7 mg for each rat) (Hebei Aierhaitai Pharmaceutical Co., Ltd., Shijiazhuang, China) was administered to the rats in group C. This study was carried out in strict accordance with the recommendations in the Guide for the Care and Use of Laboratory Animals (8th edition, 2011) of the National Institutes of Health. The animal use protocol was reviewed and approved by the Institutional Animal Care and Use Committee (IACUC) of Shandong Provincial Qianfoshan Hospital (Permit Number: 20060828001). On days 7, 14, 28 after the perfusion of bleomycin, six rats were collected from each group to obtain bronchoalveolar lavage fluid following the injection of cefotaxime sodium (600 mg/kg; Suzhou Dawnrays Pharmaceutical Co., Ltd., Suzhou, China) via the tail vein. Liquid chromatography-mass spectrometry (MS) was used to determine the concentration of cefotaxime in the lavage fluid. Lung tissues were collected for pathological observation.

### Specimen collecting and handling

A 10% chloral hydrate solution was used to anesthetize the rats by intraperitoneal injection. Following fixation, the trachea was isolated from the neck, the trachea was cut, a catheter was inserted and ligation with silk, and 2 ml saline was injected into the catheter. Following twice repeated washing, bronchoalveolar lavage fluid was collected in test tubes and centrifuged at 444 × g for 10 min. The supernatant was stored in an EP tube at −20°C. Lungs were removed and fixed with 10% formaldehyde solution until pathological examination one week later.

### Pathological examination

The right lung was dehydrated, rendered transparent and embedded in wax. The 7-μm sections were stained with hematoxylin and eosin and the pathological changes were observed under a light microscope. The extent of alveolitis and pulmonary fibrosis was evaluated according to the method of Szapiel (12). Grading standards: (−), no alveolitis or pulmonary fibrosis; (+), mild alveolitis or pulmonary fibrosis with an affected area of <20% of the whole lung; (++) moderate alveolitis or pulmonary fibrosis accounting for 20–50% of the lung; and (+++), severe alveolitis or pulmonary fibrosis with an affected area of >50%.

### Measurement of cefotaxime concentration

Chromatography was conducted using a Zorbax SB-C18 analytical column (150×4.6 mm; internal diameter, 5 μm) from Agilent Technologies (Santa Clara, CA, USA) and a 0.45-μm line filter (Agilent Technologies). The mobile phase comprised methanol and water (containing 0.1% acetic acid) in a 30:70 ratio by volume; the flow rate was 1.0 ml/min and was split to provide a flow rate of ~0.3 ml/min into a mass spectrometer (Agilent1100Trap VL-ion trap, Agilent Technologies), with an injection volume of 5 μl.

MS was conducted using an electrospray ionization (ESI) source, a heated capillary temperature of 350°C, a spray gas (N_2_) pressure of 30 psi and a drying gas (N_2_) flow of 10 l/min. The positive ion detection mode and multiple scanning with the multiple reaction monitoring (MRM) mode were used. The ionic reaction m/z 500→440 was quantitatively analyzed at a fragmentation voltage of 0.8 V.

Samples were prepared by placing 100 μl bronchoalveolar lavage fluid in a 5 ml plastic centrifuge tube, adding 400 μl methanol, vortexing for 0.5 min and centrifuging at 1,776 × g for 10 min. Then, 5 μl supernatant was used for analysis.

The specificity of the method was determined using a 10 μg/ml methanolic solution of cefotaxime sodium as the control. An ESI source was used, with a heated capillary temperature of 325°C, spray gas (N_2_) pressure of 10 psi and drying gas (N_2_) flow of 4 l/min. The perfusion injection mode was used, with a flow rate of 5 μl/min. Cefotaxime sodium mainly generated [M+Na]^+^ (m/z 500) in the positive ion mode. Selective ion scanning was used to analyze the [M+Na]^+^ peaks by fragmentation product. The most commonly observed ion fragment of cefotaxime sodium, which had an m/z ratio of 440, was considered as the product ion in the quantitative analysis.

A standard curve was generated by taking a precise amount (1.0 mg/ml) of cefotaxime sodium and adding methanol to prepare solutions of different concentrations (5, 10 and 50 μg/ml). A 5-μl aliquot of the sample solution was subjected to analysis by MS. The concentration was plotted as the abscissa and the peak area as the vertical axis of the linear standard curve to prepare a standard quantitative curve (in units of μg/ml).

### Statistical analysis

The results are expressed as mean ± standard deviation, using single-factor analysis of variance (one-way ANOVA) to analyze homogeneity of variance, ANOVA and pairwise comparisons. Statistical analysis was performed using SPSS statistical software, version 13.0 (SPSS, Inc., Chicago, IL, USA).

## Results

### Morphological changes

**Figure 1:**
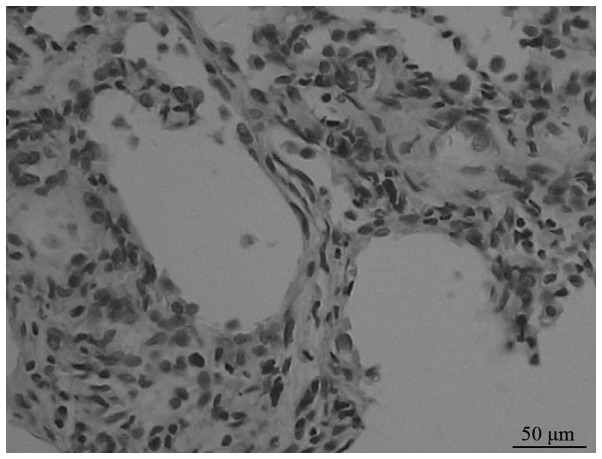
Microscopic examination of the 28-day model group (hematoxylin and eosin staining; magnification, ×400).

**Figure 2:**
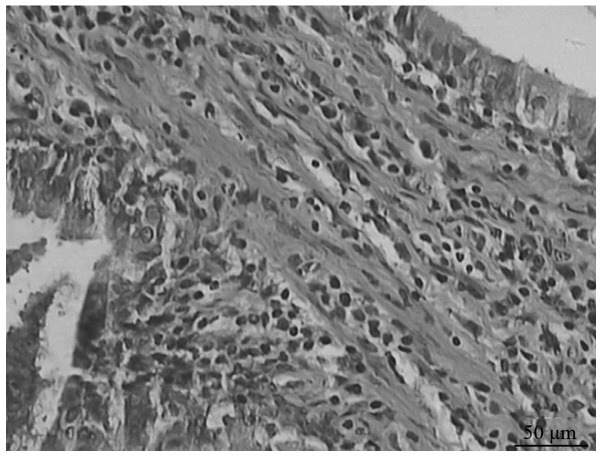
Microscopic examination of the 28-day ambroxol-treated group (hematoxylin and eosin staining; magnification, ×400).

On day 7, under a light microscope, the model group displayed moderate to severe alveolitis, but only mild alveolitis was observed on day 28. Alveolitis of the ambroxol group was mild compared with that of the untreated group. Fibrosis began from day 14 in the model group, and progressively developed until day 28, when it reached a peak (Fig. 1). The extent of pulmonary fibrosis in the ambroxol group was reduced compared with that in the model group (Fig. 2).

### Cefotaxime concentration

**Figure 3:**
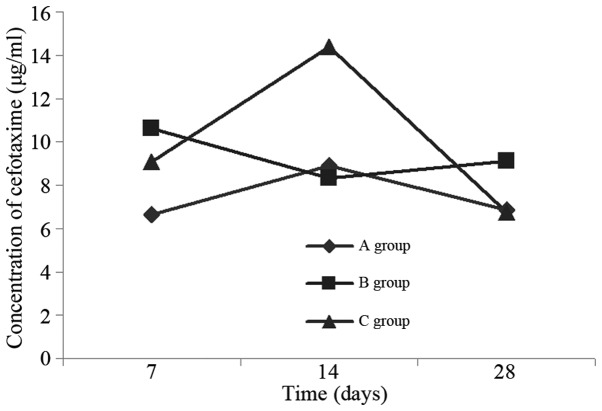
Line chart of the cefotaxime concentration in the bronchoalveolar lavage fluid at different time points in the three groups of rats.

The cefotaxime concentration in the bronchoalveolar lavage fluid of the model group was higher than that in the normal control on day 7, was reduced to a minimum on day 14, and then rose again by day 28 (Table I, Fig. 3). On day 7, the cefotaxime concentration in the bronchoalveolar lavage fluid of the ambroxol group was reduced in comparison with that in the model group; however, the difference was not statistically significant (P=0.164; Table II). On day 14, the cefotaxime concentration in the bronchoalveolar lavage fluid of the ambroxol group rose and was higher than that of the model group; the difference between the ambroxol and model groups was statistically significant (P<0.001; Table III). On day 28, the concentration in the ambroxol group dropped sharply, and was lower than that in the model group; however, the difference was not statistically significant (P=0.126; Table IV).

## Discussion

IPF complicated by infection can cause respiratory function to decline (4), exacerbate disease and increase mortality. One of the factors that is key to the success of anti-infective therapy is the concentration of antimicrobial agent in the infected tissues (9). Improving the lung tissue concentrations of antimicrobial drugs should help to control infection and improve the prognosis.

The present study aimed to determine the impact of ambroxol on the antimicrobial drug concentration in the lung tissues of rats with pulmonary fibrosis. Cephalosporins are a commonly used class of antibacterial drugs, which enter the lung tissue by diffusion mechanisms (13). In the present study, cefotaxime sodium was selected as a representative of this drug class. It was found that the concentration of cefotaxime in the bronchoalveolar lavage fluid of the model group on day7 (the alveolitis period) was significantly higher than that of the control group due to an increase of vascular permeability in the alveolitis period in the model group, which is consistent with the literature (14). The concentration in the model group had decreased by day 14 (the initial fibrosis period) and rose again on day 28 (the fibrosis stage), when no significant difference was detected when compared with the control group.

It was observed that the salt of ambroxol, in addition to improving the concentration of cefotaxime in the bronchoalveolar lavage fluid of the early fibrosis period, also reduced fibrosis, which is consistent with the literature (15,16). Previous studies have demonstrated that ambroxol has good anti-inflammatory and antioxidant effects (17–20), and can inhibit the synthesis and release of cytokines and inflammatory mediators (21). Oxygen free radical damage is an important aspect of interstitial pulmonary fibrosis. In the present study, ambroxol hydrochloride reduced alveolitis and pulmonary fibrosis. In the alveolitis and fibrosis periods, the cefotaxime sodium concentration in the bronchoalveolar lavage fluid of the ambroxol group was not significantly different from that of the model group, but was higher than that of the model group in the initial fibrosis period, suggesting that the increased impact of ambroxol hydrochloride on the cefotaxime sodium concentration in the bronchoalveolar lavage fluid in the early fibrosis period had no significant association with its anti-inflammatory and antifibrotic effects.

In conclusion, the results revealed that ambroxol hydrochloride can improve the cefotaxime sodium concentration in bronchoalveolar lavage fluid. Thus, ambroxol may improve anti-infection treatment in the early fibrosis period, and these results may act as a reference for clinical anti-infection treatment. The mechanism by which ambroxol hydrochloride increases the concentration of cefotaxime sodium in bronchoalveolar lavage fluid requires further study.

## Figures and Tables

**Table I:** Cefotaxime concentration in the bronchoalveolar lavage fluid at different time points in the three groups of rats (μg/ml).

Group	Day 7	Day 14	Day 28
A	6.64±0.32	8.90±2.48	6.85±1.08
B	10.63±2.19	8.33±2.34	9.11±4.11
C	9.09±2.14	14.39±3.21	6.74±1.63

**Table II:** Pairwise comparison of cefotaxime concentration in the bronchoalveolar lavage fluid between groups on day 7.

(I) Group	(J) Group	Mean range (I–J)	Standard error	Significant level	95% credibility interval	

					Lowest limit	Highest limit
Group B	Group A	3.9896	1.070	0.001	1.7860	6.1932
	Group C	1.5331	1.070	0.164	−0.6705	3.7368

**Table III:** Pairwise comparison of cefotaxime concentration in the bronchoalveolar lavage fluid between groups on day 14.

(I) Group	(J) Group	Mean range (I–J)	Standard error	Significant level	95% credibility interval	

					Lowest limit	Highest limit
Group B	Group A	−0.5794	1.479	0.698	−3.6249	2.4660
	Group C	−6.0588	1.479	0.000	−9.1042	−3.0133

**Table IV:** Pairwise comparison of cefotaxime concentration in the bronchoalveolar lavage fluid between groups on day 28.

(I) Group	(J) Group	Mean range (I–J)	Standard error	Significant level	95% credibility interval	

					Lowest limit	Highest limit
Group B	Group A	2.2565	1.498	0.145	−0.8290	5.3421
	Group C	2.3720	1.498	0.126	−0.7136	5.4576
